# Ultrastructural Changes in Human Trabecular Meshwork Tissue after Laser Trabeculoplasty

**DOI:** 10.1155/2015/476138

**Published:** 2015-05-12

**Authors:** Jeffrey R. SooHoo, Leonard K. Seibold, David A. Ammar, Malik Y. Kahook

**Affiliations:** Department of Ophthalmology, University of Colorado School of Medicine, 1675 Aurora Court, Mail Stop F-731, Aurora, CO 80045, USA

## Abstract

*Purpose*. To compare morphologic changes in human trabecular meshwork (TM) after selective laser trabeculoplasty (SLT) and argon laser trabeculoplasty (ALT). *Design*. Laboratory evaluation of ex vivo human eye TM after laser trabeculoplasty. *Methods*. Corneoscleral rims from human cadaver eyes were sectioned and treated with varying powers of either SLT or ALT. Specimens were examined using light microscopy, scanning electron microscopy (SEM), and transmission electron microscopy (TEM). *Results*. TEM of SLT at all powers resulted in disrupted TM cells with cracked and extracellular pigment granules. SEM of SLT samples treated at high power revealed tissue destruction with scrolling of trabecular beams. SEM of ALT-treated tissue showed increasing destruction with exposure to higher power. The presence or absence of “champagne” bubbles during SLT did not alter the histologic findings. *Conclusions*. SLT-treated human TM revealed disruption of TM cells with cracked, extracellular pigment granules, particularly at higher treatment powers. Tissue scrolling was noted at very high SLT energy levels. ALT-treated tissue showed significant damage to both the superficial and deeper TM tissues in a dose-dependent fashion. Further studies are needed to guide titration of treatment power to maximize the IOP-lowering effect while minimizing both energy delivered and damage to target tissues.

## 1. Introduction

The use of laser surgery in the treatment of glaucoma has been an option for decades, providing an alternative to topical glaucoma medications and more invasive incisional surgeries [1]. Initial efforts employed a Q-switched ruby laser to perform direct goniopuncture, although the intraocular pressure (IOP) lowering effect was short-lived [2]. Wise and Witter shifted the focus away from the concept of direct tissue puncture towards what they termed “laser trabecular tightening” with the development of argon laser trabeculoplasty (ALT) [3–5]. The Glaucoma Laser Trial (GLT) showed that initial use of ALT was at least as efficacious as treatment with the topical medications available at that time [6, 7]. In 1995, Latina and Park reported a novel technique using a Q-switched 532 nm Nd:YAG laser to perform laser trabeculoplasty [8]. Eventually termed selective laser trabeculoplasty (SLT), it was postulated to offer the advantage of selective treatment of pigmented trabecular meshwork (TM) cells without collateral thermal or structural damage.

The mechanism leading to decreased IOP after laser trabeculoplasty is unclear; proposed theories include mechanical changes in the trabecular meshwork [3] and/or induction of biologic changes at target sites, which may include trabecular cell division and migration [9] and the increased expression of cytokines and matrix metalloproteinases [10, 11]. Histopathologic evaluation of human TM has the potential to shed light on these possible mechanisms and guide the clinical use of these treatment modalities. Furthermore, the appropriate titration of laser energy during laser trabeculoplasty is poorly understood, often relying upon objective changes such as tissue whitening and bubble creation as the threshold for appropriate treatment. A previous report has shown that morphologic changes in human TM after SLT are less than those of ALT and that SLT does not result in coagulative damage [12]. In this study, we sought to further investigate ultrastructural changes in human TM after laser trabeculoplasty.

## 2. Methods

A total of three human eye bank corneoscleral rims were obtained from patients aged 74 to 82 years. In all cases, death to preservation time was less than 12 hours. All specimens had brown irides with 1-2+ trabecular pigmentation and no prior history of glaucoma. The rims were sectioned into wedges and mounted in a specimen dish using double-sided tape. The sections were kept in balanced salt solution to maintain adequate tissue hydration and then secured to the specimen dish immediately before treatment. Laser treatments were then directly applied to the TM of each specimen mounted on a platform in front of the slit lamp laser. One section was left untreated as a control. ALT was performed using a 577 nm PASCAL laser system (Topcon Medical Laser Systems, Santa Clara, CA). A 60 *μ*m spot size was used to treat at the junction of pigmented and nonpigmented TM with the pulse duration set at 0.1 seconds. Three tissue sections were treated with ALT, using powers of 300 milliwatts (mW), 600 mW, and 1000 mW. SLT was performed using a Q-switched 532 nm Nd:YAG laser (Ellex Medical Lasers, Adelaide, Australia). The spot size is fixed at 400 *μ*m, as is the duration of each laser pulse at 3 nanoseconds. Sections were treated at 0.4 millijoules (mJ), 0.5 mJ, 0.6 mJ, 0.7 mJ, 1.2 mJ, and 2.0 mJ.

After laser treatment, samples were fixed in 4% glutaraldehyde/1% formalin solution and processed for evaluation by light microscopy, transmission electron microscopy (TEM), or scanning electron microscopy (SEM). Prior to imaging with light microscopy, tissue sections were dehydrated in ethanol and embedded in EPON. Semithin sections (0.5 mm) were cut, stained with toluidine blue and sodium borate (1%), and examined by light microscopy. For SEM, tissues were fixed in 4% glutaraldehyde/1% formalin solution at 4°C overnight, dehydrated through an ethanol series, and then air-dried. Dried tissues were mounted onto metal stubs and sputter-coated with gold. Field emission scanning electron microscopy was performed on a JEOL JSM-7401F (JEOL, Pleasanton, CA). Images were recorded at 250x, 500x, and 1000x total magnification. To prepare tissue for TEM, thin tissue blocks (<1 mm thick) were dissected from the corneal rims, postfixed in 1% osmium tetroxide in 0.1 M phosphate buffer, and stained with 2% uranyl acetate. After acetone dehydration and embedding in EPON, ultrathin sections (50–70 nm) were cut and examined on a Tecnai G2 Spirit BioTWIN transmission electron microscope (FEI, Hillsboro, OR). Images were recorded at 1200x, 2400x, and 4800x total magnification.

## 3. Results

### 3.1. Untreated Specimens

**Figure 1:**
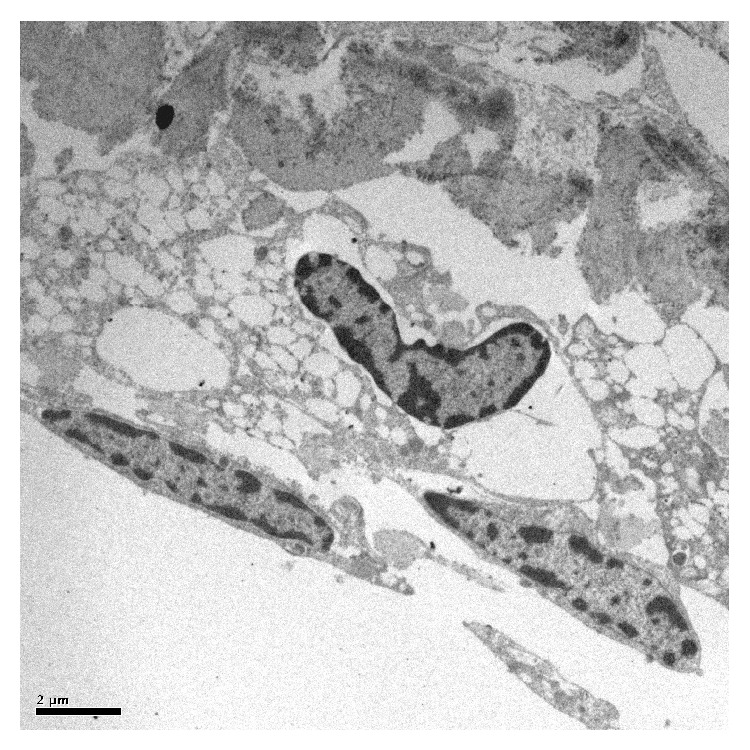
TEM of untreated TM showing intact trabecular endothelial cells and intertrabecular spaces.

**Figure 2:**
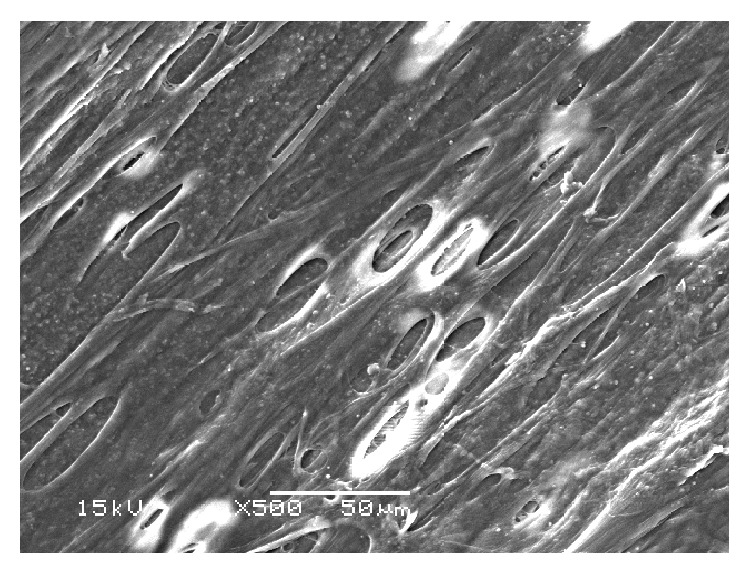
SEM of untreated tissue showing regular and intact trabecular beams.

Untreated TM examined by light microscopy revealed intact beams of trabecular tissue. TEM showed nonuniform TM structure with interspersed trabecular endothelial cells and intact pigment granules (Figure 1) and SEM revealed intact trabecular beams (Figure 2).

### 3.2. Argon Laser Trabeculoplasty

**Figure 3:**
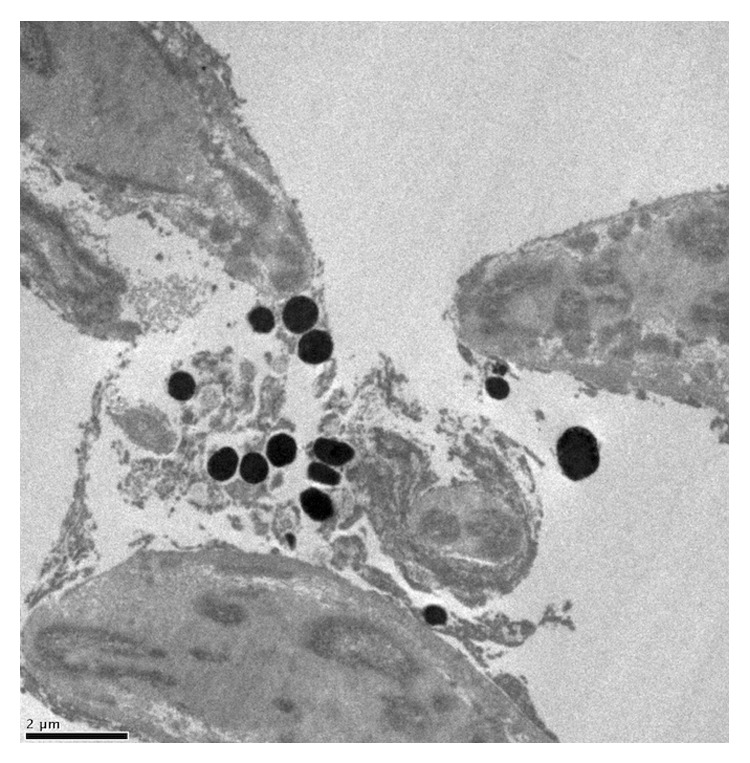
TEM of ALT-treated tissue (300 mW) showing disruption of trabecular cells with extracellular pigment granules.

**Figure 4:**
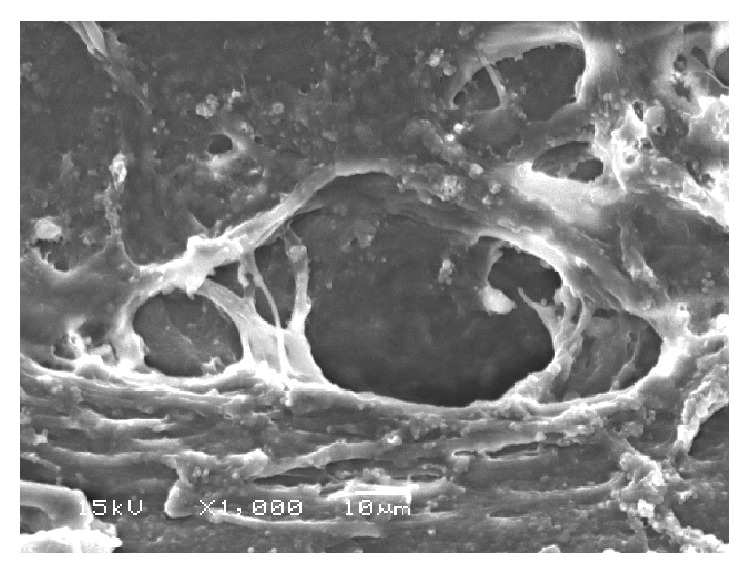
SEM of tissue treated with ALT (300 mW) showing crater formation and coagulative damage.

ALT specimens examined with light microscopy showed significant tissue disruption with loss of normal trabecular architecture. Similar findings were seen on TEM, with loss of trabecular endothelial cell integrity and dispersed pigment granules (Figure 3). SEM images showed tissue destruction with crater formation (Figure 4). Higher energy application resulted in larger craters with deeper tissue penetration.

### 3.3. Selective Laser Trabeculoplasty

**Figure 5:**
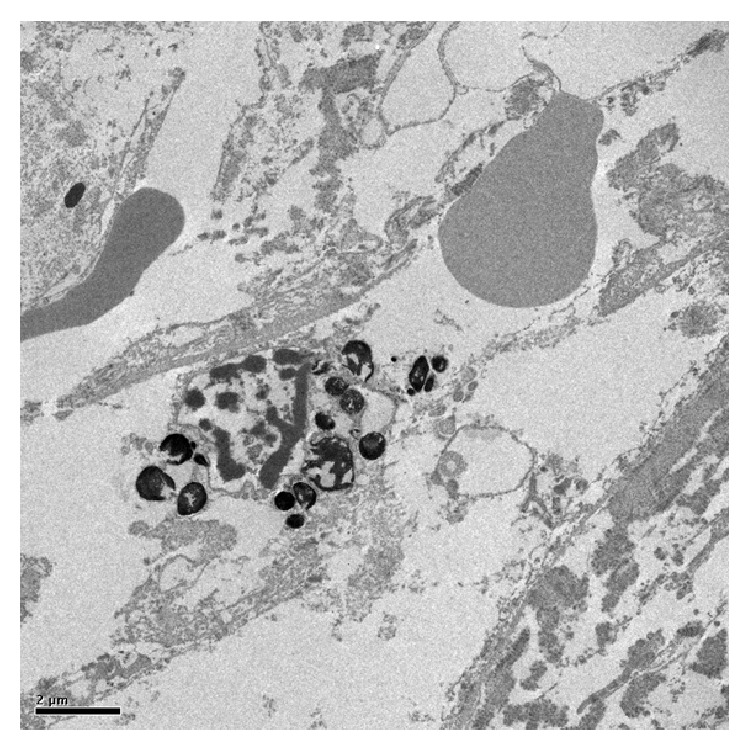
TEM of SLT-treated tissue (0.7 mJ). Extracellular, cracked pigment granules are noted.

**Figure 6:**
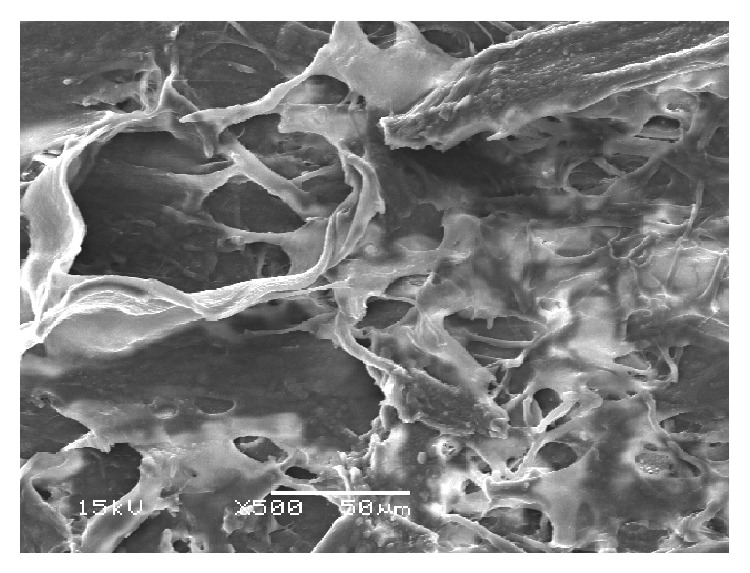
SEM of SLT-treated tissue (2.0 mJ). Note the destruction of trabecular beams with tissue scrolling at the edges of the treated areas.

Examination of SLT specimens by light microscopy showed intact trabecular beams, similar to those seen in untreated sections. TEM images showed disrupted trabecular meshwork cells with cracked extracellular pigment granules (Figure 5). These findings were consistently seen at all studied treatment powers, regardless of the presence or absence of visible “champagne” bubbles during treatment. SEM of SLT samples treated at 2.0 mJ revealed tissue destruction with scrolling of trabecular beams at the edges of the spot applications (Figure 6).

## 4. Discussion

Although laser trabeculoplasty is a well-established treatment for glaucoma, the exact mechanism of action and changes that occur in targeted tissues remains unclear. In this study, we confirm previously reported findings and report novel ultrastructural alterations in ex vivo human TM after treatment with SLT. Kramer and Noecker have shown that ALT results in coagulative damage to the TM with disruption of collagen beams and crater formation at spot application sites [12]. We confirmed these findings for ALT across a wider range of treatment powers and noted a dose-dependent response, with larger craters and more tissue destruction seen with higher-powered treatments. Our study shows that SLT at higher powers can also lead to ultrastructural damage to the TM and that the “champagne” bubble endpoint for treatment is likely not indicative of actual tissue response to treatment.

Cracked pigment granules noted by TEM after SLT have been noted previously [12], and this finding is not surprising given the purported selective treatment of pigmented cells with SLT. The lack of thermal tissue disruption seen with ALT has led to the belief that SLT may be safely repeated after initial treatment. Although prior reports have suggested that SLT does not cause disruption to trabecular beams, our findings demonstrate that SLT at higher powers is indeed capable of inducing substantial ultrastructural changes to human TM that is readily visible by SEM. It is noted, however, that these changes were only seen at a treatment power of 2.0 mJ, which is higher than that often used in clinical practice. The clinical relevance of these findings remains to be elucidated but suggests that the theory that SLT is nondestructive is inaccurate. Rather, it may be more appropriate to state that SLT is less damaging than ALT to TM tissue, particularly at lower power settings.

These findings also provide some insight regarding the mechanism by which laser trabeculoplasty lowers IOP. The mechanical theory proposes that laser energy leads to tissue contraction, which thereby opens TM and widens Schlemm's canal. Although this may play a role in increased outflow facility after ALT, it is unlikely to be the source of IOP lowering after SLT given the level of structural changes in TM noted on SEM with most of the studied SLT treatment powers. The biologic theory, on the other hand, argues that laser energy stimulates cellular expression of cytokines and increases macrophage recruitment. Previous work supports the notion that cellular repopulation of the TM may occur after trabeculoplasty, possibly due to an increase in DNA replication stimulated by laser treatment [9]. Our results suggest that the biologic theory is a more plausible explanation, although whether the amount of visible tissue damage correlates in either a positive or a negative fashion with this mechanism is unclear.

Cavitation or “champagne” bubbles released from the anterior chamber angle after a SLT laser pulse is a common sign used in clinical practice to titrate treatment power. Practitioners often titrate treatment to the lowest power at which these cavitation bubbles are noted. In our samples, these bubbles first appeared at powers of 0.6–0.7 mJ. Electron microscopy revealed no difference in findings between samples based on the presence or absence of these bubbles, although it is noted that there may be biologic effects that are not visible histologically. Given the lack of histologic differences in our samples, there is a possibility that increasing power until these bubbles are seen may falsely induce the treating physician to apply more energy to the TM than is necessary, leading to damage to cells and the loss of the potential selective nature reported with use of the SLT approach. It has been proposed that lower energy settings may be sufficient to achieve the desired effect while minimizing damage to the TM. One study comparing SLT using conventional energy (0.6–1.0 mJ) to low energy (0.3–0.5 mJ) reported no difference in postoperative IOP or success rates [13]. The low energy group also had less pain, anterior chamber reaction, and IOP spikes. Others, however, have reported more success with high-energy treatments while still using “champagne” bubbles as a treatment endpoint [14].

A major limitation in our study is the inability to correlate findings from treatment of ex vivo tissue with clinical scenarios. Direct laser application, as opposed to treatment through the cornea while the TM is surrounded by aqueous humor in the anterior chamber, may cause dissimilar effects on tissue due to differences in laser fluence and heat dispersion. Patient-specific variables, such as the degree and distribution of pigment within the TM, play an undetermined role in determining outcomes after laser trabeculoplasty. It is possible that certain populations, such as those with pigment dispersion or pseudoexfoliation, may have a greater susceptibility to damage from SLT at lower energy settings but this requires further study. Since these experiments were performed in eye bank eyes, the possibility that these findings may differ in living tissue is noted. However, a prior study of ALT and SLT performed in human eyes prior to enucleation reported fragmentation of trabecular beams with both treatment modalities using TEM for analysis [15].

Laser trabeculoplasty, either with ALT or with SLT, is a common option utilized for the treatment of glaucoma. Although the mechanism by which either approach causes IOP lowering is unclear, it is likely that a combination of changes within the TM, either mechanical or biological, leads to increased outflow facility and therefore lowering IOP. The degree of IOP lowering and duration of effect are quite variable between patients, for reasons that remain unknown. The overall safety profile for laser trabeculoplasty is excellent, but complications do occur, including postlaser IOP spikes or PAS formation after either ALT or SLT [16], and rarely, corneal edema or hyphema [17, 18]. Based on our findings, we suggest that practitioners consider lower powers for SLT and use caution when considering repeated treatments, especially if performed at higher powers. The formation of “champagne” bubbles during SLT should not be used as a metric for energy titration. Further controlled studies with clinical correlation would be beneficial to determine ideal treatment parameters in different patient populations.
